# Dietary salt intake and kidney function in rural Senegalese populations: a cross-sectional study

**DOI:** 10.1186/s41043-024-00542-2

**Published:** 2024-06-26

**Authors:** Ndongo Modou, Lot Nehemie Motoula Latou, Toure Maimouna, Amadou Diop Dia, Sidy Mohamed Seck

**Affiliations:** 1 Nephrology and Dialysis, Regional Hospital of Kedougou, Kedougou, Senegal; 2 Nephrology and Dialysis, Clinique Urgences 24, Saly, Senegal; 3 Department of Physiology, Faculty of Medicine, University Cheikh Anta Diop, Dakar, Senegal; 4 Nephrology Department, Faculty of Health Sciences, University Gaston Berger, Route de Ngalléle, BP 234, Sanar, Saint-Louis, Senegal; 5 IRL-3189 ESS/UGB/CNRS/UCAD/CNRST/USTB, Faculty of Medicine, University Cheikh Anta Diop, Dakar, Senegal

**Keywords:** Salt intake, Chronic kidney function, Epidemiology, Senegal

## Abstract

**Introduction:**

High salt intake is a major risk factor for hypertension and its complications such as chronic kidney disease (CKD) and cardiovascular diseases. The present study aimed to determine level of sodium consumption and its relation with kidney function in the rural populations of Ferlo (centre of Senegal).

**Subjects and methods:**

We performed a cross-sectional study including 400 volunteers aged > 18 years. Clinical, biological and dietary data were collected during household visits. Daily sodium intake was measured in the 24 h-urine outpout and CKD was defined as eGFR < 60 ml/min. Linear regression analysis was used to assess association between sodium intake and covariates.

**Results:**

Mean age was 46.42 ± 15.60 and sex-ratio was 1.05. Prevalence of hypertension, CKD and overweight were 21.5, 11.7 and 20.5%, respectively. The median daily salt intake was 11.7 g with interquartile range of 14.8 g. Only 11.25% of participants consumed less than 5 g/day. After multivariate analysis, high salt intake was associated with age > 60 years, overweight and CKD. However, gender and hypertension were not significantly associated with salt intake. Industrial broths (91.5%) and bread (85%) represented the main sources of dietary salt.

**Conclusion:**

This study revealed high levels of daily salt intake contrasting with low potassium intakes in the majority of participants. Participants with CKD, overweight and age > 60 years presented higher salt consumption. Stategies to reduce salt consumption are urgently needed to reduce burden of CKD in rural Senegalese populations.

## Introduction

Sodium is the main extracellular cation in the human body and plays an essential role in hydroelectrolyte and metabolic balance, particularly in the cardiovascular and neuromuscular systems [1]. Previous studies have demonstrated the link between high salt intake and several health conditions, such as chronic kidney disease (CKD), stomach cancer, and osteoporosis, and the evidence is strongest for hypertension, which in turn increases the risk of cardiovascular diseases [2, 3]. Despite the World Health Organization (WHO) warnings against high sodium intake (> 2 g/day, equivalent of 5 g of salt/day) and low potassium intake (< 3.5 g/day) the majority of adults worldwide have an average salt intake of 9 to 12 g/day [4]. In fact, poor nutrition is increasingly becoming a global health threat responsible for one fitth of deaths in 2017 [5]. Resource-limited countries are particularly affected by the lack of strong policies to reduce salt content in the diet and poor access to healthy foods [6]. Moreover, a better understanding of dietary determinants could help prevent arterial hypertension and cardiovascular diseases through more efficient strategies and actions [2, 5]. In Senegal, the prevalence of hypertension in the rural adult population was estimated to be 23.4% in 2015, and the majority of patients were not aware of their blood pressure status [7]. Poor dietary and lifestyle habits are constantly considered the main risk factors, but few studies have evaluated salt consumption [8]. The present study aimed to determine the level of sodium consumption and its relationship with kidney function in rural populations of Ferlo region (a rural area in the center of Senegal).

## Subjects and methods

### Population and sampling

We performed a cross-sectional survey using a stratified cluster sampling method to select a representative sample of the adult population. Five households were randomly selected, and in each of them, households were selected for the visits by investigating teams. After providing informed consent, volunteers aged > 18 years were included in each household to create a total sample of 400 individuals. We excluded pregnant or breastfeeding women, patients on diuretic treatment or plant species with diuretic effects, and patients with previously known renal or endocrine disease.

### Data collection

We collected sociodemographic and clinical parameters from each participant using a questionnaire. Blood samples were collected between 8 and 10 a.m. the following day, and eating habits were assessed with a 24-h dietary recall form in which the participants reported all foods and portion sizes consumed the day prior to urine collection. Daily intakes of salt from food were estimated based on proportions reported by participants and available open source tables [9]. Renal function was assessed by serum creatinine and cystatin C levels. Serum and urinary creatinine were measured using the Jaffé method. Cystatin C was measured with the colorimetric method. The glomerular filtration rate (GFR) was estimated from serum creatinine using the 4-variable MDRD equation and from serum cystatin C using the CKD-EPI equation [10]. Urinary sodium and potassium were measured with a selective electrode, and daily salt intake was estimated using the formula developed by Tanaka et al. to correct for completeness of 24-h urine collection [11]. According to 24-h natriuresis, three groups of subjects were identified: group 1, with a “normal salt intake” < 5 g/d; group 2, with a “high salt intake” between 5 and 12 g/d; and group 3, with a “very high salt intake” ≥ 12 g/d. The study protocol was approved by the ethical committee of the university (n°002019/cer/ucad).

### Statistical analysis

Data analysis was performed using the software packages Epi Info and SPSS version 18. Quantitative variables are presented as the mean with standard deviation or median with interquartile range according to the type of distribution. Qualitative variables are described as percentages. Comparisons of means, medians and proportions were performed with Student's, Mann–Whitney, chi-square and ANOVA tests, as appropriate. Pearson's linear correlation was used to detect associations in bivariate analysis. Linear regression analysis was used to assess associations between sodium intake and covariates such as age, sex, and body mass index (BMI). For all the statistical tests, we fixed the significance level at *p* ≤ 0.05.

## Results

### Sociodemographic and clinical characteristics of the population

We included a total of 400 adults comprising 205 men (51.25%) and 195 women (48.75%). The mean age was 46.4 ± 15.6 years (range 18–85 years). Overall, 62.5% of the participants were aged between 30 and 60 years. The mean BMI was 23.1 kg/m^2^ in the general population, and the prevalence of overweight/obesity was 20.5%. Table 1 presents the main sociodemographic and clinical characteristics of the participants.

**Table 1:** Sociodemographic and clinical characteristics of the participants

Characteristics	All participants	Men (n = 205)	Women (n = 195)	*p* value
Age (years)	46.4 ± 15.6	45.58 ± 17.80	47.5 ± 15.37	0.14
Age groups				0.22
< 30 years	16.0%	13.6%	18.5%	
30–60 years	62.5%	54.7%	70.7%	
> 60 years	21.5%	31.7%	10.8%	
Education level				0.09
Ileterate	57.5%	46.8%	68.7%	
Primary/secondary	42.0%	68.3%	31.3%	
University	0.5%	0.9%	0.0%	
BMI (kg/m^2^)	23.1 ± 4.8	22.92 ± 4.6	23.71 ± 5.2	< 0.01
Obesity (BMI > 30 kg/m^2^)	20.5%	17.1% 35	24.1% 47	0.25
Systolic BP (mm Hg)	133.4 ± 23.8	135 ± 24.5	131.5 ± 26.6	0.51
Diastolic BP (mm Hg)	84.7 ± 17.2	85.1 ± 13.4	84.1 ± 24.8	0.18
Hypertension	21.5%	19.0%	23.6%	0.07
eGFR1	94.9 ± 17.4	80.5 ± 12.3	77.9 ± 11.8	< 0.01
eGFR2	113.9 ± 19.5	83.7 ± 13.6	80.8 ± 12.1	0.02

*BP* blood pressure, *BMI* body mass index, *eGFR1* glomerular filtration rate estimated with a creatinine-based formula, *eGFR2* glomerular filtration rate estimated with a cystatin C-based formula

### Levels of salt and potassium intake

The median daily salt intake was 11.7 g, with an interquartile range of 14.8 g and a range between 3.64 and 28.20 g. Only 11.25% of participants consumed < 5 g of salt per day. Daily salt consumption was similar between men and women (median values of 11.65 and 11.55 g, respectively; *p* = 0.62) or between normotensive and hypertensive individuals (median values of 10.8 and 12.2 g respectively; *p* = 0.09). A positive but nonsignificant association was found between age and salt intake (r = 0.71; *p* = 0.12).

**Fig. 1:**
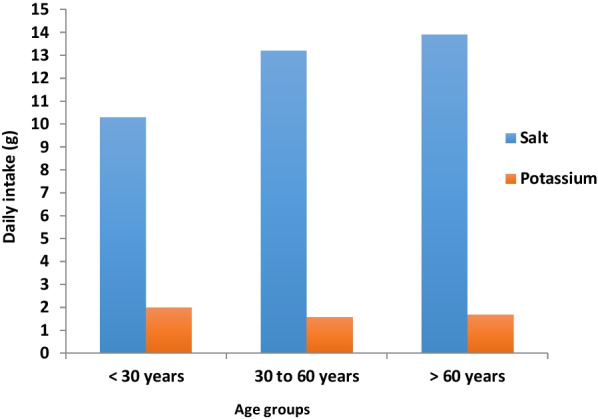
Daily salt and potassium intake across age groups

The median urinary potassium excretion was 1.5 g/d for the whole population. Similarly, men consumed significantly more potassium (1.8 g/d) than women did (1.4 g/d) (*p* = 0.03). Daily potassium intake was not correlated with age (r = − 0.13; *p* = 0.45). However, individuals < 30 years old consumed more potassium (2.0 g/d) than did those > 60 years old (1.7 g/d; *p* = 0.04). Figure 1 shows the levels of salt and potassium intake across the different age groups.

### Kidney function and sodium intake

**Fig. 2:**
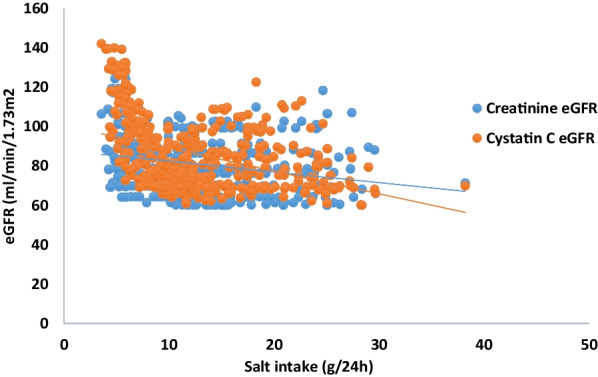
Relationship between salt intake and kidney function

Forty-seven patients (11.75%) had eGFR < 60 ml/min/1.73 m^2^. Furthermore, we found a significant correlation between salt intake and participants’ kidney function, as estimated with either serum creatinine (r = − 0.22; *p* < 0.01) or serum cystatin C (r = − 0.46, *p* < 0.01). Figure 2 shows that the eGFR decreased when sodium intake increased.

A positive and highly significant correlation was observed between BMI and salt intake (r = 0.25; *p* < 0.01). Overweight/obese participants consumed more salt than did normal weight participants (respectively 15 ± 6.48 and 11.46 ± 5.57 g/d, *p* < 0.01). Univariate and multivariate linear regression analyses revealed that age > 60 years, overweight status and low eGFR were factors associated with salt intake (Table 2). However, high blood pressure was not significantly associated with salt intake.

**Table 2:** Clinical and biological parameters associated with salt intake

	Univariate analysis		Multivariate analysis	
	β	*p* value	β	*p* value
Age > 60 years	0.21	0.01	0.15	0.03
Gender	0.13	0.57	0.18	0.20
Education level	0.06	0.11	0.03	0.12
Overweight	0.25	< 0.01	0.21	0.01
High blood pressure	0.13	0.18	0.04	0.10
eGFR1 < 60 ml/min	0.40	< 0.01	0.35	< 0.01
eGFR2 < 60 ml/min	0.25	< 0.01	0.22	< 0.01

*eGFR1* glomerular filtration rate estimated with a creatinine-based formula, *eGFR2* glomerular filtration rate estimated with a cystatin C-based formula

Sources of sodium

The common sources of dietary salt reported by participants were industrial broths and bread (Table 3). In addition, 42.5% of them admitted that they had a tendency to add salt to food during mealtimes.

**Table 3:** Sources of dietary salt and mean daily intakes reported by participants

Type of food	Number of participants	Percent (%)	Mean salt daily intake from food (g)
Industrial broth	365	91.2	6.4
Bread	340	85.0	5.7
Table salt	170	42.5	4.2
Peanuts	137	34.2	2.8
Salty chips	109	27.2	2.5
Canned food	82	20.5	1.9

Only few patients (3%) had meals out of their houses and none of them had ever started a diet to reduce salt consumption

## Discussion

This study is the first in Senegal to assess individual salt consumption in a community using 24-h urine sodium excretion. This method is considered the gold standard for the measurement and monitoring of sodium intake, as it offers a better understanding of the impact of dietary salt on nutritional status [1, 11]. We found that salt intake ranged from 3.6 to 30.2 g/day, with more than 4 out of 5 individuals taking > 5 g/day recommended by the World Health Organization (WHO) [4]. One nutritional study by Ndao Diao et al. [8] in 2020 used measurements of salt content in common dishes consumed by populations in two urban cities and estimated that salt consumption was between 8.8 and 32.3 g/day among Senegalese adults. However, previous studies demonstrated that dietary methods might overestimate salt consumption compared to urinary methods, particularly in sub-Saharan African settings where determination of food composition and portions can be challenging [5]. Globally, the average salt intake is estimated to be 10.8 g/day, but this value is highly variable [6]. In African populations, salt intake is supposed to be high, but high-quality data are scarce [12]. A survey of Morocans among students aged 18–25 years revealed lower sodium (3.1 g/day) and potassium (1.8 g/day) intakes [13]. In Ghana [14] and Benin [15], the average salt intake was 8.3 g/d and 10.2 g/day, respectively.

On the European continent, a recent systematic review reported daily salt intakes varying between 5.0 and 18.5 g in men and between 4.3 and 16.1 g in women, with lower averages in the northern and western European countries than in central and eastern Europe [16]. Other national studies reported lower sodium intake in England (8.1 g/day) [17], Latvia (8.8 g/day) [18], and Spain (9.8 g/day) [19].

A recent report from the American continent estimated that the mean salt intake in adults was 8.5 g/day, with variability ranging from 6.7 g/day in Barbados to 11.8 g/day in Colombia [20]. Similarly, studies conducted in Asia confirmed both the globalization of salt overconsumption and the disparities across populations and over time [13]. In India, reported dietary salt intake varied between 8.6 g/24 h in the North and 9.46 g/24 h in the South [21]. The average daily salt consumption is 12.7 g in South Korea [22], 11.0 g in China [23] and 10.6 g in Japan [24].

Such differences between and within continents are largely explained by the propensity of disadvantaged populations to eat more accessible salty foods. This was highlighted in a meta-analysis that included 51 studies in which individuals with a low socioeconomic status showed a 14% higher daily salt intake compared to those with a high socioeconomic status [25]. Another determinant of population behavior is the lack of knowledge about the health effects of salt. In Senegal, as in many African countries, the most popular recipes are very rich in salt, spices and fats [26]. In our study, 85% of participants admitted to regularly using industrial canned food that was rich in salt, and 42.5% admitted to systematically adding table salt before eating. However, studies evaluating the sociological determinants of people’s preference for salty foods are scarce. A good understanding of such determinants would help to define better strategies to reduce salt consumption in populations. A qualitative study of the GHANES population reported that 91% of adults admitted to a tendency to add more salt to their foods, 31.3% were unaware of the health risks associated with high salt intake, and 74.9% were convinced that they consumed the correct amount of salt [27]. These figures highlight the deleterious impact of ignorance on dietary and culinary habits in sub-Saharan Africa.

Nevertherless, variability in salt intake amounts reported in different population can also result from methodological differences between studies as discussed above.

In the present study, daily salt consumption was comparable between normotensive and hypertensive participants. Several studies have clearly demonstrated a direct relationship between salt intake and blood pressure and recognized salt restriction as one of key public health measures to fight hypertension among populations [2, 3]. The lack of significant association between blood pressure and salt intake in multivariate analysis could be explained by the low proportion of hypertensive patients among our subjects.

The major determinants of salt intake identified were age ≥ 60 years, overweight status and low eGFR. An increase in dietary salt consumption with age was also reported by Sugiuria et al. in Japan [28] and this could be explained by a physiological decline in salt taste in elderly people [29, 30]. However, the majority of data showed an inverse relationship between 24 h-natriuresis and age [31, 32].

Overweight was independently associated with a higher salt among our study subjects. This relationship between salt intake and weight gain has been previously reported in many studies [33–35]. A large multinational study showed that each 1 g/day of salt intake was associated with an increase in BMI of 0.28 in Japan, 0.10 in China, 0.42 in the United Kingdom and 0.52 in the United States (*p* < 0.01) [36]. Similarly, a meta-analysis by Moosavian et al. demonstrated that increased salt consumption was positively associated with a higher BMI and a larger waist circumference [37]. The pathophysiological mechanism of this relationship is currently poorly understood, but many hypotheses implicate alterations in adipocyte differentiation, increased appetite induced by increasing resistance to leptin, alterations in thermogenesis normally induced by food, and high carbohydrate and lipid contents in processed products sold in shops [38–42]. In addition to inducing obesity, high salt consumption could also directly or indirectly affect kidney function through an increase in blood pressure. Many clinical studies identified sodium intake as a predictor of renal outcome, even in individuals with normal renal function and blood pressure [43–46]. A lower GFR was found in our subjects with very high salt intake and a GFR was significantly correlated with salt intake (Table 2). Several authors also highlighted a significant decline in kidney function over the years related to high salt consumption [44, 47, 48].

Furthermore, the important variability in daily salt intake between and within countries is also due to the different research methods used in studies, particularly with regard to urine collection. Reducing salt intake at the population level is considered one of the most cost-effective interventions for decreasing morbidity and mortality resulting from noncommunicable diseases. Many international guidelines strongly recommend that governments develop and implement salt reduction policies, particularly in low-income countries [49]. However, few countries have effectively acheived to decrease their population’s salt consumption [6]. In Brazil, national public health measures helped to reduce population salt intake by 40 mg/day between 2008 and 2018 [50].

Like many other countries in Africa, Senegal has not yet implemented a real policy aiming to reduce sodium intake and its negative impacts on population health despite tools provided by the WHO to measure the impacts of such interventions [49].

Limitations: Despite the use of 24 h-urine output to measure natriuresis, our study was limited by some methodological constraints. Firstly, its cross-sectional design with a one-shot measure of daily salt intake might not reflect variation between days or weeks. Secondly, the small sample size yet representative of this rural population did not include enough hypertensive patients to be able to compare their salt consumption and those of normotensive individuals.

## Conclusion

The present study is the first to measure salt and potassium consumption in a rural setting in Senegal using individual 24-h natriuresis. In contrast with the WHO recommendations, we found high levels of salt intake and low potassium intake among populations. Industrial broth and bread were the main sources of salt. High salt intake was significantly associated with older age, obesity and kidney failure. A better understanding of sodium intake determinants could help define public health strategies to tackle renal and cardiovascular diseases.

## Data Availability

The dataset supporting the conclusions of this article is available upon reasonable request by mail to the corresponding author.

## Abbreviations

BMI: Body mass index
CKD: Chronic kidney disease
WHO: World Health Organization
eGFR: Estimated glomerular filtration rate
MDRD: Modification of diet in renal disease
CKD-EPI: Chronic Kidney Disease Epidemiology Collaboration
